# Thermal Impact on the Culturable Microbial Diversity Along the Processing Chain of Flour From Crickets (*Acheta domesticus*)

**DOI:** 10.3389/fmicb.2020.00884

**Published:** 2020-05-25

**Authors:** Antje Fröhling, Sara Bußler, Julia Durek, Oliver K. Schlüter

**Affiliations:** ^1^Quality and Safety of Food and Feed, Leibniz Institute for Agricultural Engineering and Bioeconomy (ATB), Potsdam, Germany; ^2^Food4Future, Leibniz Institute of Vegetable and Ornamental Crops (IGZ), Großbeeren, Germany

**Keywords:** MALDI-ToF MS, steaming, product quality, edible insects, process water

## Abstract

The role of insects for human consumption has lately increased in interest and in order to deliver safe and high-quality raw materials and ingredients for food and feed applications, processing of insects is a major pre-requisite. For edible insects a thermal treatment and appropriate storage conditions are recommended to minimize the microbiological risk and the impact of processing methods on the microbial contamination needs to be considered and determined. Based on standard process conditions for the production of *Acheta domesticus* flour, different heating treatments were used to reduce the microbial load of *A. domesticus*. In addition, the drying temperature and drying time were varied to determine whether the required residual moisture of <5% can be achieved more quickly with consistent microbial quality. The influence of the process conditions on the microbial community of *A. domesticus* along the processing chain was finally investigated under optimized process conditions. The total viable count was reduced from 9.24 log_10_ CFU/g_DM_ to 1.98 log_10_ CFU/g_DM_ along the entire processing chain. While Bacillaceae, Enterobacteriaceae, Enterococcaceae, and yeast and molds were no longer detectable in the *A. domesticus* flour, Staphylococcaceae and mesophilic spore forming bacteria were still found in the flour. The results indicate that the steaming process is essential for effectively increasing microbial safety since this processing step showed the highest inactivation. It is recommended to not only evaluate the total viable count but also to monitor changes in microbial diversity during processing to ensure microbial safety of the final product.

## Introduction

The use of insects as food poses potential microbiological risks, as insects can be vectors for human, animal, or plant pathogenic microorganisms. In 2015, the European Food Safety Authority (EFSA) estimated in its risk profile the microbiological risk for the production and consumption of insects as food and feed to be comparable to other unprocessed animal protein sources when conventionally authorized feed is used as insect substrate. In addition to insect microbiota and feed microbiota, microbial contamination of insects may also occur through production and processing conditions and during storage (FASFC, 2014; EFSA, 2015). Despite the insect species itself, the substrate and hygienic conditions during farming and processing influence the microbiological risk of edible insects (van der Fels-Klerx et al., 2018). For example, a thermal treatment and appropriate storage conditions are strongly recommended for edible insects before placing them on the market (FASFC, 2014; NVWA, 2014). Extensive studies on the presence of pathogenic microorganisms in insects used as food and feed are lacking and to protect consumers against potential health hazards these data are required, especially since no specific microbiological criteria for edible insects exists up to now in the EU (Fernandez-Cassi et al., 2019).

The microbial flora of insects in general consists of *Staphylococcus*, *Streptococcus*, *Bacillus*, *Proteus*, *Pseudomonas*, *Escherichia*, *Micrococcus*, *Lactobacillus*, and *Acinetobacter* (EFSA, 2015). Among the different edible insect species, the house cricket (*Acheta domesticus*) is a promising insect species since its protein and lipid content is comparable to beef or chicken and it is an indigenous species in many European countries (Fernandez-Cassi et al., 2019). A total aerobic mesophilic count of 10^5^–10^6^ colony forming units per gram (CFU/g) was determined for *A. domesticus*. The total bacterial count consisted mainly of Gram-negative bacteria (fecal and other coliform bacteria). Gram-positive bacteria were represented by *Micrococcus* spp., *Lactobacillus* spp., and *Staphylococcus* spp. (Belluco et al., 2013). Garofalo et al. (2017) investigated different commercially available edible insects [e.g., *A. domesticus* (whole and powder)] using cultivation-dependent and cultivation-independent methods. They found a total aerobic mesophilic bacterial count of 4.1 log CFU/g and 4.5 log CFU/g for whole *A. domesticus* and 4.8 log CFU/g and 3.9 log CFU/g for *A. domesticus* powder. Enterobacteriaceae and *Clostridium perfringens* were below the detection limit of 2 log CFU/g and *Salmonella* as well as *Listeria monocytogenes* were not detectable in 25 g. Yeasts were present in whole *A. domesticus* with 4–5 log CFU/g and in the powder <2 log CFU/g. The opposite was determined for molds. The whole *A. domesticus* showed mold counts <2 log CFU/g and the powder was between 2.9 and 3.1 log CFU/g. It has been shown that there are large variations in the microbial composition between insect species.

Cricket rearing has nowadays shifted from harvesting in the wild to mass rearing (especially in Thailand) and therefore, the establishment of good farming practices and good hygiene habits as well as the implementation of standard operating procedures and HACCP concepts in the production systems of edible insects is required to ensure high level of food safety (Fernandez-Cassi et al., 2019; Melgar-Lalanne et al., 2019). In European countries the acceptance to consume insects might be enhanced if the insects are used as flours or powders, i.e., in an unrecognizable form. To overcome consumer’s concerns and to ensure product safety, adequate post-harvest technologies have to be established in the processing of edible insects (Melgar-Lalanne et al., 2019). Different processing technologies and storage conditions were tested to improve acceptability of insects and insect products as well as to increase the shelf-life of these products.

Small or middle scale cricket production facilities include in most cases the following general processing steps of the crickets. Prior to harvest, the crickets are fasting for 24 h and after harvest they are killed either by freezing, heating, or drowning. The killing step is followed by rinsing, boiling, fast cooling, freeze-drying, and storage of the packaged crickets (Fernandez-Cassi et al., 2019). Drying is the most common used technology to increase the shelf-life of foods. Whole edible insects are preferably sun-dried (Manditsera et al., 2018), freeze-dried or oven-dried (Fombong et al., 2017), and microwave-dried (Vandeweyer et al., 2017b) and subsequently crushed or pulverized to increase consumer’s acceptance (Melgar-Lalanne et al., 2019). Besides the water reduction during drying, the color and protein functionality are changed as well as lipid oxidation can occur to different extend depending on the applied drying technique. Thus, the drying method has to be chosen with respect to the final consumption form of the insects, e.g., as whole insect or as flour. The quality of mealworms in terms of protein, fat, and chitin extractability was similar for freeze-drying and oven-drying, so that the most preferred industrial drying method is oven-drying (Purschke et al., 2018). Grabowski and Klein (2017) recommended species-specific drying procedures as well as a reheating of the insects before consumption to ensure food safety, since microorganisms may only slowed down in growth during drying and may start growing again at appropriate water conditions.

This study aimed to evaluate appropriate process conditions for the production of cricket flour. Based on standard process conditions for the production of cricket flour, which includes cooking as a heating step and drying at 110°C for 8 h, different heating steps such as boiling, steaming, and autoclaving were used to reduce the microbial load of the crickets. In addition, the drying temperature and drying time were varied to determine whether the required residual moisture of <5% can be achieved more quickly with consistent microbial quality. The influence of the process conditions on the microbial community of the crickets along the processing chain was finally investigated under optimized process conditions aiming to gain knowledge about possible resistant microorganisms and possible pathways of cross-contaminations during processing. Additionally, the microbial loads of the cricket process water as well as the protein content were evaluated to monitor the contamination of the process water.

## Materials and Methods

### Processing Chain Cricket Flour

House crickets (*A. domesticus*) were purchased from Terra-Discount (Germany) and inactivated by freezing at −20°C directly after delivery. Prior to the experiments, the crickets were thawed at room temperature for 1 h. Each process chain was conducted in three independent experiments. For better comparability a single batch of approx. 2000 g was used for all experiments.

#### Washing

Washing was conducted in three steps to remove adherent dirt and feed residues. Crickets were washed with tap water in a ratio of 7:20 for 5 min under stirring. The crickets were removed from the water using a sieve and after draining for 1 min washed in a second bath (cricket to water ratio was 7:20) for 5 min under stirring. After draining for 1 min another washing step was conducted as described before. The temperature of the used tap water before washing was 22.8 ± 0.1°C. During the first washing step the water temperature decreased to 11.6 ± 0.7°C due to the low temperature of the crickets and during washing step two and three the water temperature was 20.5 ± 0.6°C and 22.2 ± 0.5°C, respectively.

#### Thermal Treatments

After washing different thermal treatment steps (autoclaving, boiling, and steaming) were tested to reduce the microbial load of the crickets. The different thermal treatments as well as the considered treatment parameters were chosen because they were reported as conventionally applied thermal treatments (on industrial scale) for crickets in Thailand (personal communication).

Autoclaving was performed in a laboratory autoclave (Systec VX-75, Systec GmbH, Germany) for 15 min at 121°C and 2.1 bar. 100 g crickets were placed in a 250 ml beaker. The dwell time at 121°C for 15 min was ensured by placing the temperature sensor of the autoclave in the middle of the cricket samples. Three beaker containing 100 g crickets each were autoclaved at the same time.

For the boiling step, tap water was heated to 100°C in a cooking pot and crickets were then added for 10 min in a cricket to water ratio of 1:5. The temperature of the water and the crickets during boiling was recorded using a thermocouple (Model PDT300, VWR International GmbH, Germany). The crickets were cooled on ice after boiling. The boiling treatment was conducted in triplicate.

For the steaming step, crickets were placed on a gauze cloth covering a steaming insert in a cooking pot after boiling of the water. During the thermal treatment by steaming, the crickets did not lie in one layer in the steaming insert but on top of each other. After 10 min of steaming the crickets were removed and cooled on ice. The temperature of the water and the crickets during steaming was recorded using a thermocouple (Model PDT300, VWR International GmbH, Germany). Each steaming step was performed in triplicate. The statistical significance of differences between thermal treated samples was evaluated using Welch’s unequal variances *t*-test with significance levels of 0.05, 0.01, and 0.001.

#### Recording of Drying Kinetics

Drying kinetics were recorded during oven-drying (Heratherm OMH180, Thermo Electron LED GmbH, Germany) at different temperature time profiles. The temperature of the crickets during drying was recorded using a thermocouple (Model PDT300, VWR International GmbH, Germany). A drying time of 8 h at 110°C was reported as standard drying procedure for crickets in Thailand (personal communication) to ensure microbial safety and to obtain a residual moisture content <5%. In comparison to this standard drying procedure it was evaluated if residual moisture content <5% could be realized within a shorter drying time at 110°C or at lower temperatures (90°C) while ensuring microbial safety. Therefore, crickets were dried up to 4 h at 110°C and at 90°C. In addition, it was investigated whether the drying time is strongly influenced by a single-layer or multi-layer position of the crickets. After 0.5, 1, 2, 3, and 4 h, the moisture content was gravimetrically determined by differential weighing during drying at 110°C for 24 h. The TVC count was evaluated as described in section “Microbiological Analyses.” The statistical significance of differences between samples was evaluated using Welch’s unequal variances *t*-test with significance levels of 0.05, 0.01, and 0.001.

#### Pulverization

Dried crickets were pulverized to flour using a mill (Retsch Grindomix, Retsch GmbH, Germany) for 5 s at 10000 rpm.

### Color Measurements

The color of food products is an important quality parameter for consumer’s acceptance of the product. Therefore, the color of the cricket flour was determined after drying at 90°C and 110°C using a Minolta spectrophotometer (CM-2600D, Konica Minolta Inc., Japan) with CIELab system, illuminant D65, SCE (specular component excluded) mode, and 10° observer angle. The browning index (BI) was calculated using the following equations (Saricoban and Yilmaz, 2010):

(1) $$BI=\frac{\left(100\times \left(x-0.31\right)\right)}{0.17}$$

(2) $$x=\frac{a^{*}+1.75\times L^{*}}{5.645\times L^{*}+a^{*}-3.012\times b^{*}}$$

Additionally, the hue angle was recorded with 0°, red; 90°, yellow; 180°, green; and 270°, blue (McLellan et al., 1995) allowing to distinguish between intermediate colors between adjacent pairs of basic colors. All measurements were performed at three different places on the bags surface. The statistical significance of differences between samples was evaluated using Welch’s unequal variances *t*-test with significance levels of 0.05, 0.01, and 0.001.

### Microbiological Analyses

Microbiological analyses of the crickets were conducted using European standard methods. Untreated crickets, washed crickets, heat treated crickets, dried crickets, and cricket flour were prepared according to EN ISO 6887-4:2017 with slight modifications. Before homogenization of the samples using a bag mixer (BagMixer^®^ 400 CC^®^, Interscience, France) at speed 2 for 2 min and buffered peptone water as dilution medium (1:10), the crickets were coarsely crushed in the homogenization bag. In preliminary experiments the microbial load of untreated (frozen) crickets and dried crickets using a resuscitation step or without a resuscitation step was compared. As well as for untreated crickets and dried crickets no significant differences were found between the two preparation steps (data not shown). However, since the European Standard EN ISO 6887-4:2017 recommends the resuscitation step for dried samples, dried crickets and cricket flour were left in buffered peptone water for 30 min at room temperature before homogenization in the bag mixer. The aerobic mesophilic total viable count (TVC) was conducted for all samples in duplicate from each initial dilution. To evaluate the culturable microbial diversity changes during processing of cricket flour, the viable count of mesophilic spore-forming bacteria, *E. coli*, Enterobacteriaceae, *Enterococcus*, *Staphylococcus*, *Bacillus cereus*, *Clostridium perfringens*, and yeasts and molds using the same initial dilution as for the TVC as well as the presence of *Salmonella*, *Listeria monocytogenes*, and *Campylobacter* was determined along the optimized processing chain. The determination of the presence of *Salmonella*, *Listeria monocytogenes*, and *Campylobacter* required specific enrichment steps and were conducted according to European standard methods. Table 1 summarizes the applied microbiological methods. The viable count is expressed as log_10_ colony forming units per gram dry matter [log_10_ CFU/g_DM_] and each value is the average of six single values. The dry mass and the moisture content of the crickets were gravimetrically determined by drying at 110°C for 24 h.

**TABLE 1:** European and German standards applied for the evaluation of viable cell counts with corresponding growth parameters.

Target	Norm resp. analytical method	Culture medium	Growth temperature [°C]	Growth time [h]	Growth conditions
Aerobic mesophilic total viable count (TVC)	DIN EN ISO 4833-2:2013	Plate count agar (PCA)	30	72	Aerobic
Spore-forming bacteria	DIN EN ISO 6887-1:1999	Nutrient agar (NA)	37	24	Aerobic
*E. coli*	ISO 16649-2:2001:2001-04	Tryptone bilex X-glucurronide (TBX)	37 + 44	4 + 20	Aerobic
Enterobacteriaceae	EN ISO 21528-2:2009-12	Violet red bile dextrose agar (VRBG)	37	24	Aerobic
*Enterococcus* spp.	BVL L 06.00-32:1992-06	Kanamycin asculin azide agar (KAA)	37 + RT	24 + 24	Aerobic
*Staphylococcus* spp.	IS0 6888-1:1999/Amd.l:2003 (E)	Baird-Parker agar (BP)	37	48	Aerobic
*B. cereus*	EN ISO 7932:2005-03	Polymyxin pyruvate egg-yolk mannitol–bromothymol blue agar (PEMBA)	30	24	Aerobic
*C. perfringens*	ISO 7937:2004-08	Tryptose sulfite cycloserine agar (TSC)	37	20	Anaerobic
Yeasts and molds	ISO 21527-1:2008-07	Dichloran-bengal red-chloramphenicol agar (DRBC)	25	120	Aerobic
*Salmonella* spp.	EN ISO 6579:2007-10	Xylose-lysine deoxycholate agar (XLD); Brilliant green agar (BGA)	37	24	Aerobic
*L. monocytogenes*	EN ISO 11290-1:2005-01	Polymyxin acriflavine lithium chloride ceftazidime aesculin mannitol agar (PALCAM), Brilliance^TM^ Listeria	37	24–48	Aerobic
*Campylobacter* spp.	DIN EN ISO 10272-1:2017-09	Campylobacter selective agar (CCDA); Skirrow agar	42	44	Microaerophilic

In addition, the TVC of the tap water, the process water from all washing steps as well as the steaming water was examined in order to obtain a statement on the degree of contamination. Therefore, the water was serially diluted using a peptone salt solution and the dilutions were spread on plate count agar. After incubation for 72 h at 30°C the colony forming units per ml (log_10_ CFU/ml) were determined. The statistical significance of differences between samples was evaluated using Welch’s unequal variances *t*-test with significance levels of 0.05, 0.01, and 0.001.

### MALDI-ToF MS Analysis

For both the crickets and the process water, the microorganisms grown on the culture media were identified by MALDI-ToF MS (Matrix assisted laser desorption/ionization time of flight mass spectrometry) to evaluate the microbial diversity of the most abundant microorganisms. Due to the high number of different growth media and the resulting large number of grown colonies, an attempt was made to provide the best possible image of the different colonies to obtain the best possible summary of the microbial diversity. It was ensured that all colonies with different appearance were selected for further analyses. In the case of the selective media, all typical colonies were additionally examined using MALDI-ToF MS. Colonies were designated as typical if they complied with the descriptions of the European standards or the manufacturer’s specifications for the growth media. For the analyses, cell material of the colonies was suspended in 300 μl purified water and incubated for 5 min after addition of 900 μl ethanol (96%). Afterward the samples were centrifuged at 12400 × *g* for 3 min. The supernatant was removed and the samples were centrifuged again at 12400 × *g* for 1 min and the pelleted material was dried below the laminar flow and stored at −80°C until further analysis. After thawing of the samples, the pelleted material was suspended in 7.5 μl formic acid and 7.5 μl acetonitrile. Again, the samples were centrifuged at 12400 × *g* for 3 min. One μl supernatant was transferred to a target and after drying overlaid with 1 μl α-cyano-4-hydroxy cinnamic acid (CHCA) matrix (RIPAC-LABOR GmbH, Germany) and analyzed by MALDI-ToF MS (Axima Confidence, Shimadzu Deutschland GmbH, Germany) after drying. The spectra were measured using the linear mode in a mass range between 3000 and 20000 m/z and a laser repetition rate of 50 Hz. Calibration was conducted using *E. coli* ribosomal proteins. Reference mass spectra from the AnagnosTec SARAMIS^TM^ database (Spectral Archive And Microbial Identification System, bioMérieux Deutschland GmbH, Germany) were used for the identification of the obtained mass spectra. UPGMA clustering was conducted using the peak based similarity coefficient ‘Dice’ within the BioNumerics software (Version 7.6, Applied Maths NV, Belgium) using the following parameters: linear tolerance = 500 ppm and constant tolerance = 3 m/z. Reliable and unreliable clusters were separated using the cophenetic correlation. A match of the measured mass spectrum with the reference mass spectrum and a confidence level >90% led to identified clusters whereas a confidence level between 75 and 89.9% only allowed a cluster identification to the family level and a confidence level below 75% resulted in ‘not identified’ clusters. The relative abundance of microorganisms was only calculated for the identified microorganisms.

### Soluble Protein Content

The amount of soluble proteins contained in the wash and steaming water was determined via Bradford assay (Bradford, 1976) using the Roti^®^-Nanoquant Protein quantitation microassay (CarlK880, Carl Roth GmbH, Germany). Bovine serum albumin (Fluka Chemie AG, Switzerland) was used as the calibrating protein at a concentration of 1–20 μg/ml in 2 mg/ml intervals in DI water. The assay consisted of 800 μl of the sample solutions reacting with 200 μl of Bradford reagent. OD_595_ (BioPhotometer plus, Eppendorf AG, Germany) of the standard solutions and samples was measured after 20 min (20°C) against the zero value. Bradford assay was conducted in triplicates for each wash and steaming water sample. The statistical significance of differences between samples was evaluated using Welch’s unequal variances *t*-test with significance levels of 0.05, 0.01, and 0.001.

## Results and Discussion

### Thermal Treatment

The processing of cricket flour as ingredient for food products requires the reduction of microorganisms to ensure the microbial safety of the final product. A cricket core temperature of 100°C was reached after 2 min of boiling whereas during steaming the core temperature of the crickets did not reach 100°C within 10 min of steaming time. Core temperatures >95°C were reached after ∼6 min during steaming. Caparros Megido et al. (2017) achieved a core temperature of 100°C in house crickets after 3.16 ± 1.04 min of boiling and therefore set their blanching time to 4 min for house crickets. With this treatment they achieved a TVC reduction of ∼3.6 log CFU/g. It was shown that fresh and also smoked insects need a cooking step before consumption with species-specific treatment times (Caparros Megido et al., 2017). In our study, the TVCs of the thermal treated crickets were significantly reduced from ∼8 log_10_ CFU/g_DM_ to < 2 log_10_ CFU/g_DM_ by boiling, steaming, and autoclaving (Table 2). Similar results were obtained by Klunder et al. (2012) with a TVC reduction from 7.2 log CFU/g to 1.7 log CFU/g after boiling of crickets for 5 min. Comparing the TVC after boiling with the TVC after steaming or autoclaving revealed a significantly higher TVC for the boiled crickets. The moisture content of the crickets before thermal treatment was between 69 and 70% (Table 2). Autoclaving did not change the moisture content whereas steaming and boiling increased the moisture content of the crickets. An increase in moisture was also reported for mealworms after blanching (Azzollini et al., 2016). The water uptake of mealworms after blanching was attributed to water absorption and entrapment below the chitinous exoskeleton (Melgar-Lalanne et al., 2019). However, boiling led to higher moisture content in comparison to steaming. Higher moisture content might enhance the required drying time of the crickets. Since the moisture content as well as the TVC were not significantly different between autoclaved and steamed crickets, steaming was chosen as preferred method for microbial inactivation before drying and milling because steaming is easier to implement industrially than autoclaving.

**TABLE 2:** TVC and moisture content of crickets after different thermal inactivation steps.

Thermal treatment	TVC of untreated crickets [Log_10_ CFU/g_DM_]	TVC of treated crickets [Log_10_ CFU/g_DM_]	Moisture content of untreated crickets [%]	Moisture content of treated crickets [%]
Boiling	8.42 ± 0.17^a^	1.69 ± 0.04^a^	70.38 ± 1.03^a^	79.68 ± 1.74^a^
Autoclaving	8.55 ± 0.08^a^	<1.52*^b^	70.35 ± 1.96^a^	69.68 ± 1.08^b^
Steaming	8.79 ± 0.52^a^	1.56 ± 0.02^b^	68.83 ± 1.71^a^	72.39 ± 1.39^b^

*Below the detection limit. Letters in the columns represent significant differences at the significance level p < 0.001.

### Optimization of Cricket Oven-Drying

During processing of cricket flour including steaming as inactivation process and subsequent oven-drying at 110°C for 8 h the moisture content was reduced from 72% to <1% (Table 3). Kamau et al. (2018) showed that cricket flour with moisture content <5% can be stored for 7 month at 25°C. The TVC of the crickets was reduced to 1.55 log_10_ CFU/g_DM_ by steaming and further decreased to 1 log_10_ CFU/g_DM_ after drying. After milling, the TVC slightly increased to 1.39 log_10_ CFU/g_DM_ but the increase was not significant. Higher TVC values after pulverizing of crickets were also found by Fernandez-Cassi et al. (2019) and these higher values were attributed to the better extraction of microbes from the insect guts due to the pulverizing process. According to Adámek et al. (2018) mealworm, lesser mealworm, field cricket, and migratory locust should be boiled, dried at 103°C for 12 h and hermetically packaged to achieve a long-term storage of the product. Lower temperatures might lead to increased drying times and higher temperatures might decrease the required drying time.

**TABLE 3:** TVC and moisture content of crickets along the processing chain of cricket flour (thermal treatment: steaming; oven-drying: 110°C, 8 h).

Sample	TVC [Log_10_ CFU/g_DM_]	Moisture content [%]
Untreated crickets	8.96 ± 0.80^a^	74.25 ± 7.49^a^
Washed crickets	8.27 ± 0.13^a^	72.29 ± 7.74^a^
Steamed crickets	1.55 ± 0.09^b^	71.57 ± 5.72^a^
Dried crickets	1.00 ± 0.00^c^	0.68 ± 0.49^b^
Cricket flour	1.39 ± 0.60^b,c^	0.72 ± 0.24^b^

*Letters in the columns represent significant differences at the significance level p < 0.001.*

**FIGURE 1:**
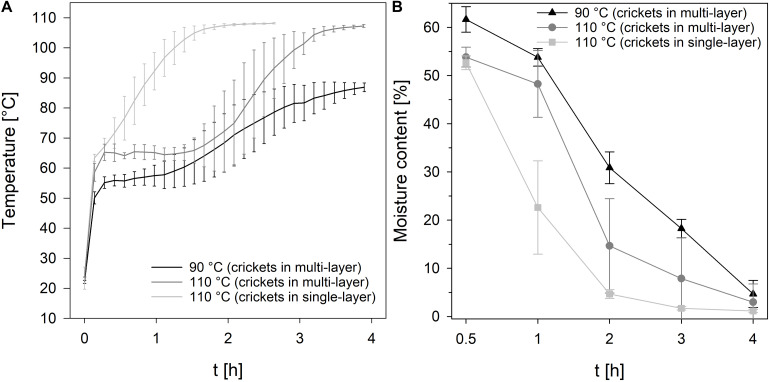
Temperature profiles **(A)** and moisture content **(B)** of crickets during oven-drying at 90°C and 110°C.

**FIGURE 2:**
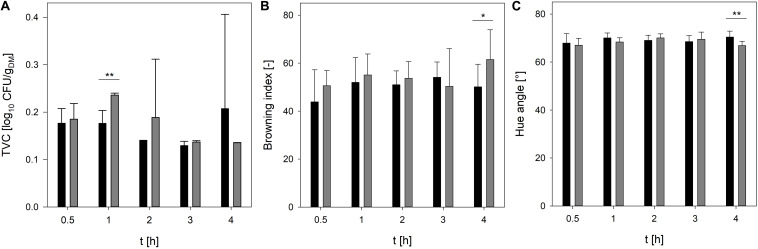
TVC **(A)**, browning index **(B)**, and hue angle **(C)** of crickets during oven-drying at 90°C (black bars) and 110°C (gray bars). Statistical significances were calculated between the different drying temperatures at the different drying times with *p < 0.05 and **p < 0.01.

To reduce the process time, in particular the drying time, crickets were dried in single-layer and multi-layer and the moisture content, TVC, and color of the crickets were recorded during drying at 90°C and 110°C for 4 h. As expected, the temperature profile of the crickets revealed that the core temperature of the crickets increased much slower if the crickets were dried in multi-layer in comparison to the drying of crickets in single-layer (Figure 1A). At 90°C, the temperature of the crickets reached 86°C and at 110°C a core temperature of 107°C was reached after 4 h of drying crickets in multi-layer. In contrast, drying of crickets in single-layer increased the core temperature of the crickets within 1.8 h to 107°C. A moisture content of 4.7% and 3.0% was reached after 4 h multi-layer drying at 90 and 110°C, respectively (Figure 1B). Especially the multi-layer drying at 110°C showed high standard deviations revealing inhomogeneous drying of the crickets. At 110°C and drying of crickets as a single-layer, a moisture content of 4.7% was already achieved after 2 h and after 3 h the moisture content was reduced to 1.7%. Since the applied steaming process effectively reduced the microbial load of the crickets, microbial contamination of the crickets was monitored during drying, especially to detect recontamination during the process. Traditionally applied sun-drying processes for edible insects showed a recontamination of the insects due to contact with soil as well as poor hygiene and storage conditions (Caparros Megido et al., 2017). Only minor changes in the TVC of the crickets were determined during drying at 90°C and 110°C (Figure 2A) indicating that no recontamination occurred during the drying process. With the exception of 1 h drying, no significant differences (*p* < 0.01) in the TVC were found between drying at 90°C and 110°C indicating that both temperatures are suitable for maintaining the achieved microbial reduction during steaming.

Since color changes are the most visible quality parameters during drying (Melgar-Lalanne et al., 2019), the L^∗^a^∗^b^∗^ values as well as the hue angle of crickets dried at 90 and 110°C were determined and the browning index was calculated. At both temperatures, the browning index was not significantly changed during drying (Figure 2B). Additionally, there was no significant difference (*p* < 0.05) between the browning index at 90 and 110°C with the exception for the longest drying time of 4 h. At 110°C and 4 h drying time the browning index was significantly higher than at 90°C and the same drying time. The increase in the browning index can be attributed rather to non-enzymatic browning reactions than to enzymatic browning reactions (Azzollini et al., 2016). The results imply that at higher temperatures non-enzymatic browning is more pronounced than at lower temperatures. Hue angles of ∼70° were recorded for the crickets dried at 90 and 110°C whereas the drying time did not influence the hue angle (Figure 2C). However, similar to the browning index, a drying time of 4 h showed a significant lower hue angle of the crickets dried at 90°C in comparison to 110°C indicating again a higher browning of the crickets.

The results reveal that the drying time of crickets in single-layer can be reduced to 3 h at 110°C and still moisture content below 5% is realized while maintaining the achieved microbial inactivation during steaming and without further affecting the color of the crickets.

### Microbial Community Structure Along the Processing Chain of Cricket Flour

Based on the results of the thermal treatment experiments and the optimization of cricket oven-drying an experimental process chain for the production of cricket flour was defined. The production chain of cricket meal comprises a three-stage washing step, followed by thermal treatment by steaming for 10 min and oven drying at 110°C for 3 h before the crickets are ground. A health threat to consumers results in particular from the contamination and proliferation of unexpected pathogens in the food supply chain. Food batches are often sampled for specific microorganisms, so that unexpected pathogens can go undetected. Fernandez-Cassi et al. (2019) introduced a risk profile for house crickets as a novel food and identified data gaps on the impact of thermal processing of the products. Since no specific microbiological criteria are available in the EU up to now, it is proposed to use microbiological criteria of minced meat for edible insects (Caparros Megido et al., 2017). However, the consumption of whole insects, including the intestinal tract, makes it difficult to comply with the microbiological criteria for minced meat (Fernandez-Cassi et al., 2019). Garofalo et al. (2019) conducted a literature review about the microbiota of both fresh or processed edible insects and revealed market variations in microbial load and diversity as well as species-specific microbiota of the edible insects. However, most of the studies are dealing either with fresh or with processed insects and only few studies are available on the microbial dynamics during production (Stoops et al., 2017; Vandeweyer et al., 2018; Wynants et al., 2018). Appropriate HACCP systems for the supply chain of edible insects have to be established (Garofalo et al., 2019). The investigation of microbial changes along the processing chain could play an important role in the implementation of microbiological criteria. But the development of bacterial contaminants in interaction with various process sequences is currently not sufficiently investigated. The question of whether an inadequate inactivation measure can also lead to an increased proliferation of human pathogens in the further course of the product chain and thus cause serious cases of illness when the infectious dose is taken up is also not answered yet. For this reason, a characterization of the microbial diversity on the surface of crickets during processing was carried out. Table 4 summarizes the viable count obtained on the different selective and non-selective growth media along the processing chain of cricket flour. Similar viable counts of the untreated crickets on the selective growth media in comparison to the TVC were found on the specific growth media for *Enterococcus*, *Bacillus cereus*, and *Clostridium perfringens* while the specific growth medium for spore-forming bacteria showed the lowest viable count. These results were also obtained after the steaming process but after steaming the viable count of spore-forming bacteria was higher than the TVC. Klunder et al. (2012) also revealed higher counts of spore-forming bacteria after heat treatments and suggested an activation of the spores due to the heat treatment. After drying for 3 h at 110°C growth of microorganisms was only detected on the non-selective growth medium and on the selective growth media for *Staphylococcus* and *B. cereus*. After pulverization, growth of microorganisms was again detected on the selective growth media for *B. cereus*, *Enterococcus*, *Staphylococcus*, *C. perfringens*, spore-forming bacteria, and yeasts and molds. The increased microbial load after pulverization is in accordance with other studies and is attributed to the release of gut microbiota (Garofalo et al., 2019).

**TABLE 4:** Total viable counts on non-selective and selective growth media along the processing chain of cricket flour.

Growth media	Untreated crickets [Log_10_ CFU/g_DM_]	Washed crickets [Log_10_ CFU/g_DM_]	Steamed crickets [Log_10_ CFU/g_DM_]	Dried crickets (110°C, 3 h) [Log_10_ CFU/g_DM_]	Cricket flour [Log_10_ CFU/g_DM_]
PCA [Aerobic mesophilic total viable count]	9.24 ± 0.38^a^	8.51 ± 0.48^b^	2.83 ± 0.42^c^	1.75 ± 0.25^d^	1.98 ± 0.28^d^
TBX [*Escherichia coli*]	6.77 ± 0.25^a^	5.91 ± 0.34^b^	<2.10*^c^	<1.63*^d^	<1.63*^d^
VRBG [Enterobacteriaceae]	8.01 ± 0.75^a^	7.10 ± 0.35^b^	<2.10*^c^	<1.63*^d^	<1.63*^d^
PEMBA [*Bacillus cereus*]	9.33 ± 0.33^a^	8.53 ± 0.35^b^	2.61 ± 0.55^c^	1.64 ± 0.02^d^	1.88 ± 0.35^d^
KAA [*Enterococcus*]	9.01 ± 0.45^a^	8.20 ± 0.40^b^	<2.10*^c^	<1.63*^d^	1.65 ± 0.02^d^
TSC [*Clostridium perfringens*]	9.16 ± 0.44^a^	8.19 ± 0.65^a^	2.42 ± 0.38^a^	<1.63*^a^	1.64 ± 0.01^a^
BP [*Staphylococcus*]	8.62 ± 0.70^a^	7.70 ± 0.53^b^	2.45 ± 0.40^c^	1.64 ± 0.02^d^	1.70 ± 0.14^d^
DRBC [Yeasts and moulds]	6.45 ± 0.33^a^	5.62 ± 0.48^b^	<2.10*^c^	< 1.63*^d^	1.64 ± 0.01^d^
NA [Aerobic mesophilic spore-forming bacteria]	4.14 ± 1.61^a^	3.91 ± 1.81^a^	3.54 ± 2.09^*a,b*^	<1.63*^b^	1.70 ± 0.14^b^

*Below the detection limit. Letters in the rows represent significant differences at the significance level p < 0.05.

**FIGURE 3:**
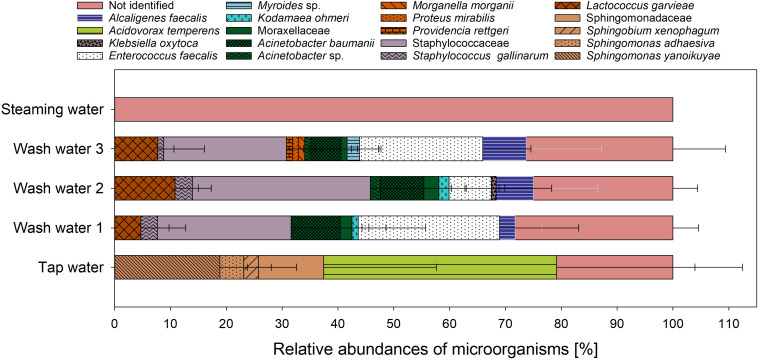
Relative abundances of analyzed microorganisms from crickets along the processing chain of cricket flour.

The occurrence of foodborne pathogens such as *Salmonella*, *Listeria monocytogenes*, and *Campylobacter* in crickets seems to be low (Walia et al., 2018), however, the presence of pathogens should be excluded. Along the processing chain, growth of microorganisms on selective growth media for *Salmonella* and *L. monocytogenes* occurred in all tested samples whereas growth on selective media for *Campylobacter* occurred only in untreated and washed crickets (Table 5). The growth of microorganisms on special selective growth media is not necessarily associated with the presence of potentially pathogenic microorganisms. For the verification of suspected pathogenic microorganisms further investigations of the grown microorganisms are necessary. Suspected microorganisms on selective growth media for *Staphylococcus*, *Salmonella*, and *L. monocytogenes* were found in all samples whereas suspected microorganisms for *Campylobacter* and *C. perfringens* were only found in untreated and washed crickets. *Enterococcus* suspected microorganisms were found in untreated and washed crickets as well as in the cricket flour. *B. cereus* suspected microorganisms were only found in washed crickets and in the cricket flour. The identification of these suspected microorganisms via MALDI-ToF MS analysis revealed that none of them could be clearly identified as the suspected foodborne pathogen, even though the SARAMIS^TM^ database contains 957 *Staphylococcus* spectra, 269 *Salmonella* spectra, 246 *Listeria* spectra, 299 *Campylobacter* spectra, and 697 *Clostridia* spectra. The comparison with the SARAMIS^TM^ database either did not lead to an identification of the microorganisms or they were identified as Enterobacteriaceae, *Klebsiella oxytoca*, *Klebsiella pneumoniae*, *Proteus mirabilis*, *Pseudomonas aeruginosa*, *Citrobacter* sp., *Enterobacter* sp., Staphylococcaceae, or *Kodamaea ohmeri*. Only suspicious *Enterococcus* colonies found in untreated and washed samples as well as in cricket flour using selective media for Enterococcaceae could be identified as *Enterococcus faecalis* (data not shown). While in the untreated and washed crickets about 8 log_10_ CFU/g_DM_ *E. faecalis* were found using the selective media for Enterococcaceae, the amount of *E. faecalis* in cricket flour was reduced to 1.7 log_10_ CFU/g_DM_ (data not shown). Even if the typical colonies on the selective media could not be identified as *Salmonella*, *Listeria monocytogenes*, *Staphylococcus* sp. or *Campylobacter*, potentially human pathogenic microorganisms can be found among the microorganisms identified instead. These results clearly emphasize the necessity to identify grown microorganisms in order to verify the presence of pathogenic microorganisms. The results show that there are not only special groups of microorganisms on the food samples, but that in principle unexpected potential pathogens can also occur. The detection of such unexpected pathogens is of considerable importance, particularly with regard to tailored concepts of hygienic procedures and proper decontamination techniques preventing risks to consumers’ health. Traditionally, the identification of the bacterial species after cultivation on the growth media is conducted via morphological, physiological, and biochemical characterization which is very time consuming. In this context, MALDI-ToF MS is a rapid, accurate, and cost-effective technique which has shown to achieve significantly better species identification than conventional biochemical methods or 16S rRNA sequencing (Böhme et al., 2012). To obtain an overview of the culturable microbial community structure of the crickets along the processing chain of cricket flour, grown microorganisms on the selective and non-selective growth media were analyzed by MALDI-ToF MS. Altogether, 1123 grown colonies were analyzed for the untreated crickets, 1091 colonies for the washed crickets, 267 colonies for the steamed crickets, 157 colonies for the dried crickets, and 147 colonies for the cricket flour. The comparison of the obtained mass spectra with the reference mass spectra of the SARAMIS^TM^ database and subsequent cluster analysis led to 35.1 ± 3.4% unidentified microorganisms for the untreated crickets, 41.8 ± 6.1% unidentified microorganisms for the washed crickets, 24.9 ± 24.1% unidentified microorganisms for the steamed crickets, 39.0 ± 38.2% unidentified microorganisms for the dried crickets, and 49 ± 34% unidentified microorganisms for the cricket flour (data not shown). The high number of unidentified microorganisms, which is also reported in other studies for different samples (Hausdorf et al., 2013; Rahi et al., 2016; Fröhling et al., 2018), can be the result of spectra with poor quality or can be caused by the occurrence of unknown microorganisms. Another explanation can be the lack of reference spectra for environmental samples especially for insects since most commercially available databases mainly include clinically relevant microorganisms (Welker and Moore, 2011; Rahi et al., 2016). The relative abundance of analyzed culturable microorganisms is presented in Figure 3. Microorganisms belonging to ten different families were identified for the untreated crickets. Members of the family Enterobacteriaceae were the most common microorganisms on the untreated crickets, followed by members of the families Enterococcaceae, Streptococcaceae, Moraxellaceae, Staphylococcaceae, Morganellaceae, Metschnikowiaceae, Pseudomonadaceae, Bacillaceae, and Paenibacillaceae. Vandeweyer et al. (2017a) also found Enterobacteriaceae, Pseudomonadaceae, Enterococcaceae, and Streptococcaceae in whole crickets using metagenetic analysis. After washing, eleven families were detected with relative abundances in descending order: Enterobacteriaceae, Streptococcaceae, Moraxellaceae, Pseudomonadaceae, Enterococcaceae, Morganellaceae, Metschnikowiaceae, Staphylococcaceae, Brucellaceae, Bacillaceae, and Paenibacillaceae. Steaming of the crickets reduced the number of families to five: Enterobacteriaceae, Bacillaceae, Enterococcaceae, Morganellaceae, and Streptococcaceae with relative abundances in descending order. Enterobacteriaceae was also the most predominant family on dried cricket samples followed by Micrococcaceae, Morganellaceae, Staphylococcaceae, Enterococcaceae, Metschnikowiaceae, Moraxellaceae, and Bacillaceae, i.e., the number of detected families increased after drying to eight families. Osimani et al. (2017) also detected Moraxellaceae in dried crickets using PCR-DGGE techniques and Garofalo et al. (2017) found Pseudomonadaceae and Staphylococcaceae in dried crickets using metagenetic analysis. In both studies also Enterobacteriaceae and Bacillaceae were detected in the dried crickets. The cultivable microorganisms from the cricket flour were composed of seven families, whose relative frequencies decreased in increasing order: Enterobacteriaceae, Moraxellaceae, Morganellaceae, Bacillaceae, Enterococcaceae, Staphylococcaceae, and Streptococcaceae. For all tested samples the family Enterobacteriaceae showed the highest diversity with four species identified and no differences were found in the samples during processing of the cricket flour. Members of the families Pseudomonadaceae, Paenibacillaceae, and Brucellaceae were only found in untreated and washed crickets implying that these microorganisms were more sensitive to the steaming and drying process than the microorganisms of the other families. In contrast, *Micrococcus luteus* who is belonging to the family Micrococcaceae was only found in the dried crickets. Osimani et al. (2017) also found members of the family Micrococcaceae in whole dried crickets but their found species did not belong to the genus *Micrococcus*. *Micrococcus luteus* can be found on human skin and can be released into air (Kooken et al., 2012) and the presence of *Micrococcus luteus* in the dried crickets might be related to contamination during the drying process. However, in the cricket flour *Micrococcus luteus* was not found but it has to be taken into account that only grown microorganisms from the highest dilution were identified. Since the viable count was slightly higher after pulverization due to better accessibility to the microorganisms it might be possible that other microorganisms were more dominantly grown on the growth media and therefore the presence of *Micrococcus luteus* might be suppressed in the other samples. Pseudomonadaceae and Paenibacillaceae were detected in cricket powder by Garofalo et al. (2017) and Osimani et al. (2018), respectively, but in both studies culture-independent methods were used in comparison to the culture-dependent method used in our study. Using 16S rRNA gene amplicon sequencing, Vandeweyer et al. (2018) showed that heat-treated tropical house crickets had a similar microbial community composition as crickets during rearing even though the viable count decreased. They suspected that not all of the DNA was destroyed and was therefore detected by the cultivation-independent analysis method applied. However, it was shown that the microbial community composition of dried tropical house crickets is comparable to the microbial community composition of live tropical house crickets. In accordance, comparable microbial community composition of the crickets during processing to cricket flour was revealed in our study. Nevertheless, it has to be noted that the abundances of the different families within the microbial composition changed during processing indicating different resistances of the microorganisms toward the applied heat and drying processes.

**TABLE 5:** Growth on selective media after enrichment procedures with indication of the growth of suspected microorganisms.

Selective media	Untreated crickets [Growth/Suspected colonies]	Washed crickets [Growth/Suspected colonies]	Steamed crickets [Growth/Suspected colonies]	Dried crickets (110 °C, 3 h) [Growth/Suspected colonies]	Cricket flour [Growth/Suspected colonies]
Selective enrichment for *Salmonella*	+/+	+/+	+/+	+/+	+/+
1^*st*^ enrichment for *Listeria*	+/+	+/−	+/−	−/−	−/−
2^*nd*^ enrichment for *Listeria*	+/−	+/−	+/−	+/−	+/−
Selective enrichment for *Campylobacter*	+/+	+/+	−/−	−/−	−/−

**FIGURE 4:**
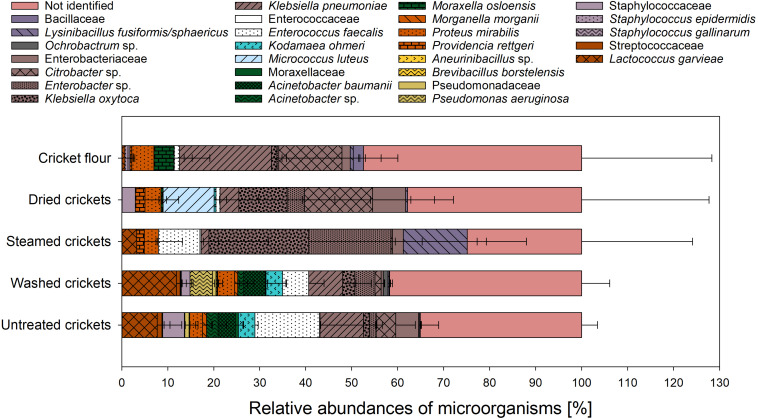
Relative abundances of analyzed microorganisms from process water along the processing chain of cricket flour.

During processing of crickets to flour a washing step was applied to remove the adherent dirt and feed substrate from the crickets. In consequence, the wash water may contain high amounts of microorganisms as well as organic matter (Gil et al., 2015). The high content of organic matter (e.g., proteins, carbohydrates, and lipids) lead to difficult disposal problems and potentially contribute to severe pollution problems (Hansen and Cheong, 2019). The microbial load of the cricket process water as well as its protein content was evaluated in this study and the results are presented in Table 6. The protein content of the cricket wash water from the first washing step was 65.6 μg/ml and in the wash water from the second and third washing step the protein contents were 27.7 and 27.8 μg/ml, respectively. The protein content of the water from the steaming process was not significant different from the protein content of the tap water. The cricket wash water had a TVC of ∼ 6log_10_ CFU/ml in all washing steps and since the used tap water had a TVC of ∼2 log_10_ CFU/ml the microbial load of the wash water is the result of the migration of microorganisms from the crickets into the water. The process water from the steaming step only had a TVC of 0.5 log_10_ CFU/ml. Wynants et al. (2017) also found an increase of the TVC in mealworm rinsing water and concluded a removal of microorganisms from the mealworm. Process water from meat, poultry, and seafood processing is characterized by a high content of proteins and fats and may contain significant amounts of pathogens (Hansen and Cheong, 2019). Since crickets are also of animal origin and microbial criteria of minced meat or seafood are suggested for edible insects, the occurrence of pathogens should also be taken into account in cricket wash water. Therefore, the grown microorganisms on the non-selective growth medium were also identified by MALDI-ToF MS to gain knowledge about the microbial community structure in the wash water (Figure 4). For the tap water 71 grown colonies were analyzed and thereof 20.8 ± 12.5% could not be identified. The identified culturable microbial community of the tap water consisted of the two families Comamonadaceae and Sphingomonadaceae whereby Comamonadaceae were composed only of *Acidovorax temperans* and Sphingomonadaceae included the genera *Sphingomonas* spp. and *Sphingobium*. In contrast, all four grown colonies from the steaming water could not be identified. The low number of microorganisms analyzed is due to the fact that the total bacterial count in the steaming water was very low. 28.3 ± 4.6% of the 88 analyzed colonies from the first wash water could not be identified and the identified microbial community consists of members of the families Staphylococcaceae, Enterococcaceae, Streptococcaceae, Alcaligenaceae, Moraxellaceae, and Metschnikowiaceae with abundances in descending order. In comparison to the first wash water, Enterobacteriaceae was additionally found in the second wash water. Altogether, 87 colonies were analyzed from the second wash water with 25.0 ± 4.4% unidentified microorganisms. Enterobacteriaceae could not be found among the 91 analyzed colonies from the third wash water, instead Flavobacteriaceae were detected. 30.3 ± 0.6 of the analyzed colonies in the third wash water remained unidentified. The results showed that the microbial community structure of the wash water is almost composed of the same microorganisms as that of the crickets whereby the relative abundances of the species were different. However, it should be noted that only the TVC was examined for the process water and that additional selective culture media were used for the crickets, leading to the possibility of a resulting shift in the relative abundances of the microorganisms. Nevertheless, Alcaligenaceae and Flavobacteriaceae were only found in the wash water and not on the crickets. This could be due to the fact that these microorganisms were found only on the surface of the insects and could therefore more easily migrate into the washing water. In consequence, these microorganisms were more common in the wash water than on the crickets and were therefore methodically found only in the wash water and not on crickets. The migration of microorganisms from the crickets into the wash water during processing has to be considered and monitored to avoid a transmission of potential pathogens from the wash water into the environment.

**TABLE 6:** Aerobic mesophilic total viable count and protein concentration of the process water along the processing chain of cricket flour.

Sample	Aerobic mesophilic total viable count [Log_10_ CFU/ml]	Protein concentration [μg/ml]
Tap water	2.21 ± 0.22^a^	0.24 ± 0.27^a^
Wash water 1	6.22 ± 0.15^b^	65.56 ± 5.26^b^
Wash water 2	6.06 ± 0.16^*b,c*^	27.69 ± 3.03^c^
Wash water 3	5.94 ± 0.18^c^	27.84 ± 2.54^c^
Steaming water	0.48 ± 0.57^d^	1.24 ± 1.58^a^

*Letters in the columns represent significant differences at the significance level p < 0.05.*

## Conclusion

It is important to ensure microbial safety in edible insects and products thereof in order to avoid risks to human health. As there are currently no valid microbiological criteria for placing edible insects on the market, the microbiological criteria for minced meat are consulted. This can be viewed critically since insects are consumed with intestinal tract and therefore the microbiota of the intestinal tract strongly increases the microbial load. The higher microbial load due to the use of whole insects results in the need for appropriate inactivation procedures to reduce the microbial load in order to ensure microbial safety. Consumer’s acceptance for the consumption of insects is higher if the insects are presented in unrecognizable form such as powder or pastes. The manufacture of such products requires suitable processes that produce a microbiologically safe product. When processing, it is important to implement and comply with hygiene regulations and to determine suitable process parameters as well as to introduce critical control points. In order to establish microbiological criteria for edible insects and the products thereof, detailed knowledge of the microbial community structure of the insects as well as the dynamic changes of the microbial community structure during processing is necessary.

The processing chain of cricket flour including steaming as inactivation step effectively reduced the TVC of the crickets which was maintained during drying and pulverization. The analysis of the culturable microbial community structure changes during processing using MALDI-ToF MS revealed that the microbial community of cricket flour is similar to the microbial community of untreated crickets but relative abundances of the microorganisms changed, indicating different sensitivities of the microorganisms against the different processing steps. While Enterobacteriaceae were the predominant microorganisms in all samples, the amount of Bacillaceae was higher in cricket flour than in the untreated crickets revealing a higher resistance of Bacillaceae against the applied inactivation treatments. However, an increased growth of potential human pathogenic microorganisms as a result of a treatment measure could not be derived from these results. Nevertheless, the influence of processing on both harmful microorganisms and endemic microflora (loss of the protective function of microbial colonization) must also be considered. The analysis of the process water revealed a migration of microorganisms into the water as well as an increase of organic matter which has to be taken into account to avoid environmental pollution and possible release of potential pathogens. From the results it can be concluded that, in addition to the total bacterial count, the composition of the microbial community should also be examined in order to ensure the microbial safety of the products. The detection of unexpected pathogens is of importance to develop tailored concepts of hygienic procedures and proper decontamination techniques preventing risks to consumers’ health.

## Data Availability Statement

The datasets generated for this study are available on request to the corresponding author.

## Author Contributions

AF conceived and designed the experiments, performed the experiments, analyzed and interpreted the data, and wrote the manuscript. SB and JD contributed to the design of experiments, performed the experiments, and proofread the manuscript. OS contributed to the design of experiments, contributed reagents, material, analysis tools or data, and proofread the manuscript.

## Conflict of Interest

The authors declare that the research was conducted in the absence of any commercial or financial relationships that could be construed as a potential conflict of interest.
